# Pomegranate flower polysaccharide improves mastitis in mice by regulating intestinal flora and restoring the blood-milk barrier

**DOI:** 10.3389/fphar.2024.1427355

**Published:** 2024-08-15

**Authors:** Jianlong Li, Wen Yin, Yuan Liang, Zhaoran Yang, Liangliang Li, Zhanhai Mai, Xingjian Yu, Yabin Lu, Zhenping Zhang, Saifuding Abula, Yi Wu, Adelijiang Wusiman, Qingyong Guo

**Affiliations:** ^1^ College of Veterinary Medicine, Xinjiang Agricultural University, Urumqi, China; ^2^ College of Veterinary Medicine, Nanjing Agricultural University, Nanjing, China; ^3^ College of Animal Science and Veterinary Medicine, Henan Agricultural University, Zhengzhou, China; ^4^ Department of Biochemistry and Molecular Medicine, School of Medicine, University of California, Davis, CA, United States; ^5^ College of Veterinary Medicine, Yunnan Agricultural University, Kunming, China

**Keywords:** *Punica granatum*, anti-inflammation effect, gut microbiota, RAW 264.7, ear swelling, paw swelling

## Abstract

This study explored the inhibitory effect of pomegranate flower polysaccharide (PFPS) on mastitis through *in vitro* and *in vivo* models. PFPS is a new type of polysaccharide isolated and extracted from pomegranate flowers. The result revealed that PFPS consists of GalA, Ara, and Gal, and the residues consist of 1,4-GalpA, 1,4-Galp, and 1,3,6-Galp, which contain HG-type and RG-I-type pectin structural domains. *In vitro* studies showed that PFPS could inhibit LPS-enhanced phagocytosis of RAW 264.7 cells and the release of IL-1β, IL-10, and TNF-α. *In vivo*, studies showed that PFPS improved xylene-induced mouse ear swelling and carrageenan-induced mouse paw edema by inhibiting inflammatory factors. In the mouse mastitis model, PFPS significantly improved LPS-induced inflammation and oxidative stress in mammary tissue. Intestinal flora sequencing results showed that PFPS could effectively regulate the intestinal flora of mice, reduce the relative abundance of pathogenic bacteria *Oscillospira* and *AF12*, and increase the probiotics *Blautia*, *Parabacteroides*, *Allobaculum*, and *Clostridiaceae_Clostridium*. Therefore, PFPS ultimately played a role in preventing mastitis by regulating the intestinal flora and further improving the blood-milk barrier. This study provides a scientific basis for PFPS as a potential candidate drug for the treatment of mastitis.

## 1 Introduction

Mastitis is a common animal disease in actual production activities, which is manifested by hard, swollen, painful and feverish udders (Sun et al., 2022). Mastitis has a serious impact on animal health and dairy products, specifically: reduced milk production, affected dairy product quality, increased breeding costs, weakened reproductive performance, and threatened public health (Hu et al., 2019). The causes of animal mastitis are complex and diverse, among which pathogenic microorganisms account for the highest proportion (Gillespie and Oliver, 2005). Currently, the most commonly used and most effective treatment is the use of antibiotics. However, due to the abuse of antibiotics, the number of drug-resistant strains has increased and drug residues have endangered public health (Hu et al., 2019). Therefore, the development of alternative drugs to antibiotics has become a research focus. Neutrophil infiltration in breast tissue is the main diagnostic criterion for mastitis (Guidry et al., 1998). The blood-milk barrier is mainly composed of mammary epithelial cells and tight junctions between cells (Tsugami et al., 2017). The increase in the permeability of the blood-milk barrier promotes the migration of neutrophils, so maintaining the blood-milk barrier is important for inhibiting mastitis (Wang et al., 2017a). Recent studies have reported that intestinal flora plays an important regulatory role in maintaining the blood-milk barrier. For example, the bacterial metabolite Lipopolysaccharide (LPS) increases the permeability of the blood-milk barrier, while short chain fatty acid (SCFA) can restore the blood-milk barrier by increasing the content of tight junction proteins (Wang et al., 2017b). Improving mastitis by regulating intestinal flora and maintaining the blood-milk barrier has become a popular research direction.

Pomegranate (*Punica granatum* L.) belonging to the Lythraceae family, is a cash crop with great economic value cultivated worldwide (Caruso et al., 2020). Although the pomegranate is more commonly regarded as a fruit, the leaves, flowers, pericarp, and roots from pomegranate shrubs have been applied in the treatment of numerous diseases, giving the pomegranate significant value as traditional medicines (Faria and Calhau, 2011; Karimi et al., 2017; Vucic et al., 2019). For example, pomegranate leaf is an important recipe in traditional Chinese medicine for hemostatic purposes, which can be used to relieve nosebleeds, external bleeding, vomiting blood, and other symptoms of the disease (Lansky and Newman, 2007; Colombo et al., 2013); the extract of pomegranate bark is reported as antibacterial agent to treat antibiotic resistant pathogens (Leesombun et al., 2022); the tannins and flavonoids from pomegranate peel show good antihyperglycemic, antihepatoprotective and antioxidant effects (Middha et al., 2013). Other researches have unveiled the therapeutic capabilities of pomegranate such as anti-inflammatory (Xu D. et al., 2022), antioxidant (Wei et al., 2022), anti-diabetic (Li et al., 2008), vasoprotective (Wang et al., 2018), and neuroprotective (George et al., 2023) functions. In general, the medicinal values of pomegranate are still being discovered and developed, and its pharmacological properties can be attributed to the enriched and diverse secondary metabolites, such as ellagic acid, punicalagin, alkaloids, and phenolics (Wibaut and Hollstein, 1957; Liu and Seeram, 2018; Hosseini et al., 2023). However, among numerous publications on *P. granatum*, few have reported the chemical structure and pharmacological activities of polysaccharide from pomegranate flower. Polysaccharides have a significant regulatory effect on the microbiota and can be converted into SCFA by the microbiota to regulate the metabolic process of animals (Aoki et al., 2021). Studies have shown that improving intestinal flora can promote the expression of tissue barrier proteins and ultimately inhibit the occurrence of tissue inflammation (Li et al., 2023; Ran et al., 2024). Ran et al. (2024) used FMT experiments to confirm that Hesperetin improves the blood-milk barrier by regulating intestinal flora to inhibit the activation of the TLR4/NF-κB signaling axis, ultimately inhibiting the occurrence of mastitis in mice. Li et al. (2023) found that Maslinic acid can promote the expression of barrier proteins in mammary tissue through intestinal flora, thereby inhibiting mastitis. Therefore, we proposed a scientific hypothesis on whether PFPS can improve the blood-milk barrier by regulating intestinal flora and ultimately inhibit mastitis.

## 2 Materials and methods

### 2.1 Materials and reagents

Dulbecco’s Modified Eagle Medium (DMEM) was purchased from Thermo Scientific; Dexamethasone (DEX) sodium phosphate injection was purchased from Shanxi Ruicheng Kelong Veterinary Drug Co., Ltd.; Lipopolysaccharide (LPS) was purchased from Sigma-Aldrich; Vybrant phagocytosis assay kit was purchased from Molecular Probes; Trizol reagent and reverse transcription kit were purchased from Life Technologies; TNF-α and IL-6 kit were both purchased from Neobioscience; IL-1β was purchased from Wuhan Boster Biological Technology., LTD.; CAT, SOD, MDA, GSH, T-AOC and MPO kit were purchased from Nanjing Jiancheng Biological Engineering Research Institute; ZDM-1101 enzyme labeler was purchased from Droide Instrument Equipment Shanghai Co., Ltd.; TGL-16A centrifuge was purchased from Changsha Pingfan Instrument and Meter Co., Ltd.; CO_2_ incubator was purchased from Thermo Fisher Scientific Co. Ltd.; TS100 inverted microscope was purchased from Nikon; FACSAria II flow cytometer was purchase from Becton, Dickinson, and Company.

### 2.2 Separation and purification of PFPS

**FIGURE 1:**
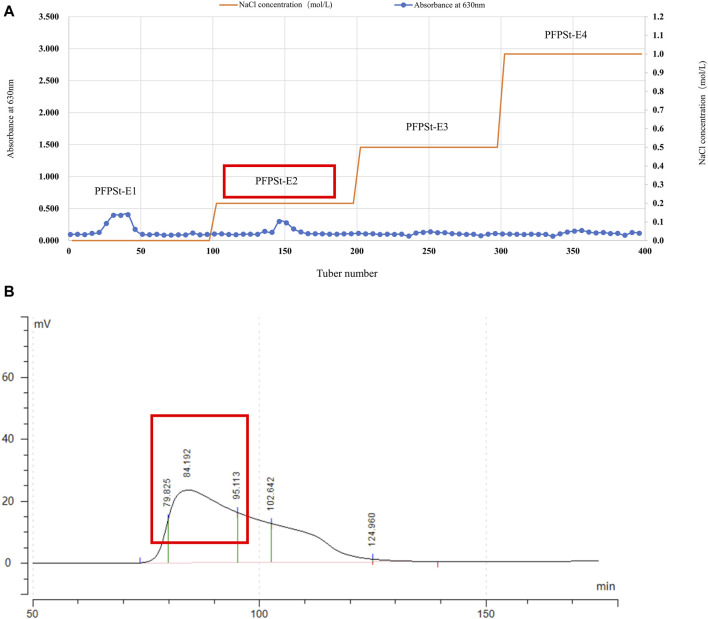
The separation of PFPS **(A)** Ion exchange column chromatography elution profiles of PFPSt **(B)** Gel column chromatography elution profiles of PFPSt-E2.

Pomegranate flower was purchased from the Second People’s Hospital of Xinjiang Uygur Autonomous Region and specimen (No. 20200302) was deposited in College of Veterinary Medicine, Xinjiang Agricultural University. The pomegranate flowers (1 kg) were refluxed with 95% ethanol (5 L) at 65°C under reduced pressure twice for 5 h each. Then, add 8 times the amount of pure water and heat for 4 h. The two filtrates were combined and 90% ethanol was added to the filtrate to give a final concentration of 80% to yield the crude PFPSt (total crude polysaccharides of Pomegranate flower) after 24 h. The crude PFPSt was dissolved in pure water and added to sevage reagent five times to remove proteins, followed by spinning under reduced pressure to remove organic reagents. Then the remaining water content was removed by lyophilization. The crude PFPSt (30 g) extract was reconstituted in water (300 mL). The resulting PFPSt solution was centrifuged at 8,000 rpm for 10 min and then filtered through a 0.45 µm microporous membrane to remove the insoluble precipitates. Then the filtrate was purified by ion-exchange chromatography column (DEAE sepharose FF) and eluted with deionized water of deionized water (dH_2_O), 0.2 M NaCl, 0.5 M NaCl, and 1.0 M NaCl successively to collect the eluted fractions. The sugar content of each eluted fractions was analized by the phenol-sulfuric acid method to give the elution peak. The experiment of phenol-sulfuric acid quantitative method was based on the method of Yue et al. (2022). dH2O and 0.2 M NaCl solution eluted more polysaccharides than 0.5 M NaCl and 1.0 M NaCl solutions. Considering that the molecular weight eluted from 0.2 M NaCl was more compatible, the 0.2 M NaCl elution fraction was selected for purification by gel filtration column chromatography (Figure 1A). Eluted fractions were performed on a gel filtration chromatography column (Chromdex 200PG), eluted with ultrapure water. Eluted fractions of the peak (80–95 min) were collected, combined, and named PFPS (Figure 1B). Then the eluate fractions were concentrated under reduced pressure and freeze-dried under a vacuum. The ion-exchange chromatography column and gel filtration column chromatography were performed using the method we reported previously (Wu et al., 2022).

### 2.3 Characterization of the PFPS

#### 2.3.1 Molecular weight of PFPS

The dextran standards (1, 5, 12, 25, 50, 80, 150, 270, 410, and 670 kDa) was diluted by 0.05 M NaCl solution to make 5 mg/mL dextran standard solutions. The molecular weight of PFPS was performed by High-Performance Gel Permeation Chromatography (Yang et al., 2023). The standard solution was added to PFPS to formulate test sample solutions with 5 mg/mL PFPS.

#### 2.3.2 Monosaccharide composition of PFPS

1 mL of 2 M trifluoroacetic acid solution was added to 5 ± 0.1 mg PFPS sample and the mixture was heated at 121°C for 2 h. Then the extracts were concentrated by centrifugation, reconstituted with sterile water, and analyzed by chromatography to determine the monosaccharide composition of PFPS. Using Chromeleon, the content of each component in the sample (µg/mg) was calculated according to the formula = C × V × F/M (C is the instrumental reading concentration, V is the volume of sample extract, F is the dilution factor, and M is the total amount of sample weighed).

#### 2.3.3 Elucidation of PFPS linkage

PFPS (10 mg) extract was dissolved in ultrapure water (1 mL), followed by addition of 100 mg/mL carbodiimide (1 mL). After 2-h incubation, the solution was divided into two groups and 2 M imidazole (1 mL) was added separately. 1 mL of 30 mg/mL of NaBH4 and 1 mL of 30 mg/mL of NaBD4 were added to each solution and the mixture was incubated for 3 h. The reaction was terminated by the addition of 100 µL of glacial acetic acid to obtain the reduced sample. The samples were dialyzed for 48 h and lyophilized. The lyophilized PFPS was dissolved in 500 µL DMSO, and 1 mg NaOH was added and incubated for 30 min. Then 50 µL of iodomethane solution (30 mg/mL) was added to methylated all hydroxyl groups, followed by 1 mL water and 2 mL dichloromethane were added, which were further centrifuged to obtain the dichloromethane phase. Subsequently, the PFPS was hydrolyzed to monosaccharides to further determine the linkage of the sugar residues. Firstly, 100 µL of 2 M TFA was added, and the reaction was carried out at 121°C for 90 min. Then, 50 µL of 2 M ammonia and 50 µL of 1 M NaBD4 were added, which was carried out at room temperature for 2.5 h. The reaction was terminated by the addition of 20 µL of acetic acid, followed by the addition of 250 µL of acetic anhydride, which was carried out at 100°C for 2.5 h. Finally, 1 mL water and 500 µL methylene chloride were added, and the dichloromethane phase was taken for detection after centrifugation.

#### 2.3.4 Nuclear magnetic resonance (NMR) analysis of PFPS

The lyophilized samples were dissolved in 0.5 mL D2O and TMSP was added as an internal standard. One-dimensional (1H-NMR and 13C-NMR) and two-dimensional (including 1H-1H COSY, TOCSY, HSQC, HMBC, and NOESY) were determined using a 600 MHz Bruker NMR spectrometer.

### 2.4 *In vitro* anti-inflammatory activity of PFPS

#### 2.4.1 Cell viability

RAW264.7 cells were purchased from American Type Culture Collection (ATCC). RAW 264.7 cells in the logarithmic growth phase were adjusted to 5×10^5^ cells/mL, cultured in 96-well plates at 100 μL per well, and placed in a 37°C, 5% CO_2_ incubator for 24 h. After discarding the culture medium in the wells, 100 μL of different concentrations of PFPS (1,000, 500, 250, 125, and 62.5 μg/mL) made from DMEM cell culture medium were added to each well with explicated experiments for each concentration. After 24 h of incubation, CCK-8 solution was used to evaluate the cell viability.

#### 2.4.2 Inflammatory factor

RAW 264.7 cells in PFPS-treated groups were cotreated with different concentrations of PFPS (62.5, 125, 250 μg/mL) and LPS (1 μg/mL) for 24 h. The Model group only used LPS (1 μg/mL) modeling. The supernatant was collected to determine the content of IL-1β, TNF-α, and IL-10 by ELISA kits.

#### 2.4.3 Phagocytic ability of neutral red

The cell processing method is the same as 2.4.2. After incubation for 24 h, add 100 μL of 0.1% neutral red to each well and continue incubation for 1 h. Rinse twice with PBS to remove excess neutral red, and finally add 100 μL cell lysate (ethanol and 0.01% acetic acid at the ratio of 1:1) to each well. After incubation at 4°C overnight, the samples were measured at the absorbance of 540 nm by a microplate reader.

#### 2.4.4 Phagocytic ability of *Escherichia coli*

The cell processing method was consistent with 2.4.2. After incubation for 24 h, the FITC-labeled *Escherichia coli* solution supplied by Vybrant phagocytosis assay kit was added to the cells and incubated for 2 h. Finally, 100 μL of Taipan blue suspension was added and the phagocytosis rate was determined by flow cytometry.

### 2.5 *In vivo* anti-inflammatory activity of PFPS

#### 2.5.1 Animals

All mice were purchased from Liaoning Changsheng Biotechnology Co., Ltd. and kept at the Laboratory Animal Center of Xinjiang Agricultural University with a standard diet and in standard specific pathogen-free (SPF) conditions between 20°C and 23°C, with 12 h light/dark cycles. Animal experiments were performed in compliance with the guidelines of the Xinjiang Agricultural University Institutional Animal Care and Use Committee (IACUC), detailed in the IACUC-approved protocol (No. 2022009). All animals were adaptively reared for 1 week before the formal experiment.

#### 2.5.2 Mouse ear Edema’s swelling test

Thirty-six Kunming mice (half females and half males, 6–8 weeks) were divided into six groups randomly: Control group, Model group, DEX group, PFPS-L group, PFPS-M group, and PFPS-H group. The Control group and Model group were gavaged with saline. The DEX group was gavaged with dexamethasone (10 mg/kg). The PFPS groups were gavaged with diverse doses of PFPS (PFPS-L: 400 mg/kg; PFPS-M: 800 mg/kg; PFPS-H: 1,600 mg/kg, 20 mL/kg). Each group was treated for 7 days. 1 h after the last administration on day 7, dimethylbenzene (0.03 mL) was smeared on both sides of the right ear of each mouse in the Model group, DEX group, and PFPS-treated groups, while mice of the Control group were treated with saline (0.03 mL) as the same method. 0.5 h after administration of the dimethylbenzene/saline, the peripheral blood of each mouse was harvested from the heart. Then the mouse was sacrificed by cervical dislocation and the whole eras of each mouse (both treated and untreated) were collected and stacked symmetrically, and then a hole puncher (8 mm in diameter) was used to collect the ear fractions. The weight of each ear fraction was tested to calculate the inhibition ratio, the swelling rate and inhibition rate were calculated as follows.

$$\text{Swelling Rate }\%=\frac{\text{Weight}\;\text{of}\;\text{Right}\;\text{ear}\;\text{fraction}-\text{Weight}\;\text{of}\;\text{left}\;\text{ear}\;\text{fraction}}{\text{Weight}\;\text{of}\;\text{left}\;\text{ear}\;\text{fraction}}\times 100\%$$

$$\text{Inhibition rate }\%=\frac{\text{Average}\;\text{swelling}\;\text{rate}\;\text{of}\;\text{inflammation}\;\text{group}-\text{Average}\;\text{Swelling}\;\text{rate}\;\text{of}\;\text{admininastration}\;\text{group}}{\text{Average}\;\text{swelling}\;\text{rat}e\;\text{of}\;\text{inflammation}\;\text{group}}\times 100\%$$

#### 2.5.3 Carrageenan-induced paw edema assay

Thirty-six Kunming mice (half females and half males, 6–8 weeks) were divided into six groups randomly: Control group, Model group, DEX group, PFPS-L group, PFPS-M group, and PFPS-H group. The Control group and Model group were gavaged with saline. The DEX group was gavaged with dexamethasone (10 mg/kg). The PFPS groups were gavaged with diverse doses of PFPS (PFPS-L: 400 mg/kg; PFPS-M: 800 mg/kg; PFPS-H: 1,600 mg/kg, 20 mL/kg). Each group was treated for 7 days. 1 h after the last administration on day 7, 1% carrageenan (50 μL) was injected into the voix pedis of the right-hind limb of each mouse in DEX, Model, and PFPS-treated groups, and the mouse in the control group was treated with saline following the same method. At the time points of 0, 1, 2, 3, 4, 5, 6, and 7 h after the injection, the thickness of the right-hind limb for each mouse was measured three times and the swelling ratio and inhibition ratio were calculated as follow.

$$\text{Swelling Rate }\%=\frac{\text{Thickness}\;\text{of}\;\text{Voix}\;\text{Pedis}\;\text{after}\;\text{injection}-\text{Thickness}\;\text{of}\;\text{Voix}\;\text{Pedis}\;\text{before}\;\text{injection}}{\text{Thickness}\;\text{of}\;\text{Voix}\;\text{Pedis}\;\text{after}\;\text{injection}}\times 100\%$$

#### 2.5.4 Construction of the mouse mastitis model

Seventy-two Kunming mice (42 females and 21 males) were divided into six groups: Control group, Model group, DEX group (5 mg/kg dexamethasone), PFPS-L group (400 mg/kg PFPS), PFPS-M group (800 mg/kg PFPS), PFPS-H group (1,600 mg/kg PFPS). Pregnant female mice were separated after 1 week of combined cage rearing. After the females gave birth, the females were separated from the pups, when the body color of the pups changed from dark red to slightly reddish. The Control and Model groups were gavaged with saline from day 7 to day 14, and the Control group used PBS modeling on day 15. The DEX group was gavaged with dexamethasone from day 7 to day 14. The PFPS groups were given diverse doses of PFPS from day 7 to day 14. On day 15, mastitis models were established in all groups except the Control group. To establish a mouse mastitis model, 0.3% sodium pentobarbital solution was injected intraperitoneally for anesthesia, 1 mm of the nipple was cut off with ophthalmic scissors to expose the milk ducts, and 50 μL of 0.2 mg/mL LPS solution was slowly injected into the mammary gland.

#### 2.5.5 Inflammatory factor and oxidative stress markers

The content of IL-1β, TNF-α, IL-10, CAT, SOD, MPO, GSH, MDA, and T-AOC in tissue was determined following the kit manual Section 2.4.2.

#### 2.5.6 HE staining and immunofluorescence

The ear and breast tissues of mice were fixed with 10% neutral formalin solution and submitted to Powerful Biology Co., Ltd. (Wuhan, China) for staining. The quantification was performed by ImageJ.

#### 2.5.7 16S rRNA sequencing analysis

Fresh colon stool samples frozen in liquid nitrogen were submitted to Personalbio Biotechnology Co., Ltd. (Shanghai, China) for 16S rRNA sequencing analysis. Experimental results are uploaded to the GenesCloud Platform for data analysis.

#### 2.5.8 qPCR

Trizol was used for RNA extraction reagent followed by reverse transcription into cDNA using the provided kit as per the manufacturer’s instructions. SYBR Green PCR Master Mix was employed for conducting qPCR following the instructions provided by the manufacturer. The primer sequences used are listed in Table 1.

**TABLE 1:** Primers for qRT-PCR.

Symbol	Primer sequences (5′-3′)
Mouse-TNF-α	Forward: CAC​CAC​GCT​CTT​CTG​TCT​ACT​GAA​C
	Reverse: AGA​TGA​TCT​GAG​TGT​GAG​GGT​CTG​G
Mouse-IL-6	Forward: ACT​TCC​AGC​CAG​TTG​CCT​TCT​TG
	Reverse: TGG​TCT​GTT​GTG​GGT​GGT​ATC​CTC
Mouse-IL-1β	Forward: CAC​TAC​AGG​CTC​CGA​GAT​GAA​CAA​C
	Reverse: TGT​CGT​TGC​TTG​GTT​CTC​CTT​GTA​C
Mouse-GPX3	Forward: AAC​GTA​GCC​AGC​TAC​TGA​GGT​CTG​A
	Reverse: CCA​CCT​GGT​CGA​ACA​TAC​TTG​AGA​C
Mouse- GPX1	Forward: TTC​CCT​CAA​GTA​CGT​CCG​ACC​TG
	Reverse: TCT​CAC​CAT​TCA​CTT​CGC​ACT​TCT​C
Mouse-SOD2	Forward: TCC​CAG​ACC​TGC​CTT​ACG​ACT​ATG
	Reverse: CTC​CTC​GGT​GGC​GTT​GAG​ATT​G
Mouse-CAT	Forward: CCA​TAG​CCA​GAA​GAG​AAA​CCC​ACA​G
	Reverse: GGA​ATC​CCT​CGG​TCA​CTG​AAC​AAG
Mouse-Occludin	Forward: ACG​GAC​CCT​GAC​CAC​TAT​GA
	Reverse: TCA​GCA​GCA​GCC​ATG​TAC​TC
Mouse-Claudin-3	Forward: CCA​CCA​TTG​GCA​TGA​AGT​GC
	Reverse: AGA​GGT​TGT​TTT​CCG​GGG​AC
Mouse-Actb	Forward: GTG​ACG​TTG​ACA​TCC​GTA​AAG​A
	Reverse: GCC​GGA​CTC​ATC​GTA​CTC​C

### 2.6 Statistical analysis

All results are expressed as the mean ± standard deviation, graphed by GraphPad Prism 6.0 software for ANOVA multiple comparisons. *P* < 0.05 is considered statistically significant in this analysis.

## 3 Result

### 3.1 The characterization of PFPS

**FIGURE 2:**
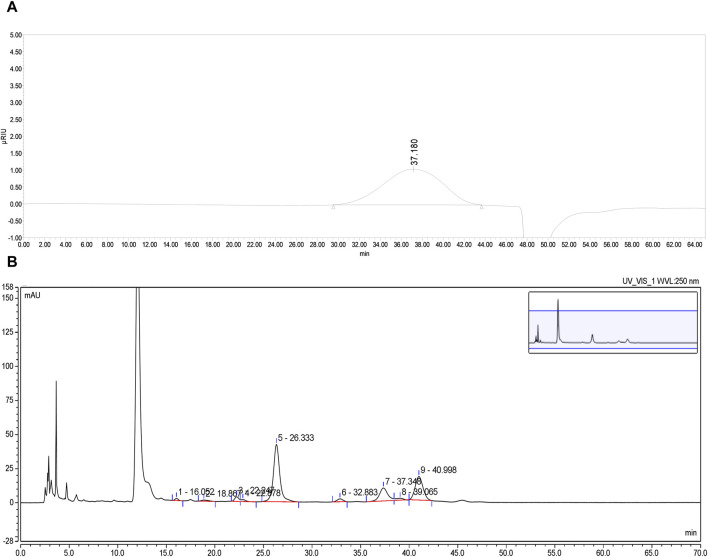
**(A)** The HPGPC spectrum of PFPS. **(B)** HPLC chromatograms of PFPS hydrolyzed derivatives.

PFPS is a polysaccharide extracted and purified from Pomegranate flowers. The HPGPC detection result showed a single symmetrical peak (37.18 min), so PFPS was a purified polysaccharide with a single composition (Figure 2A). By comparing the chromatograms with those of 11 monosaccharide standards, it was determined that PFPS was mainly composed of Galacturonic Acid, Arabinose, Galacto-ose and Rhamnose (Figure 2B). The molecular weight and physicochemical chemical composition of PFPS summarized in Table 2.

**TABLE 2:** The molecular weight and physicochemical chemical composition of PFPS.

Sample PFPS	
Mp (kDa)	29.139
Mw (kDa)	36.250
Mn (kDa)	24.285
Monosaccharide compositions (%)	
Galacturonic Acid (GalA)	56.735
Arabinose (Ara)	20.631
Galactose (Gal)	12.020
Rhamnose (Rha)	5.267
Glucose (Glc)	1.550
Glucuronic Acid (GlcA)	1.341
D-Glucosamine hydrochloride (GlcN)	1.020

The results of the methylation analysis mainly contained 1,4-GalpA sugar residues and contained 1,4-Galp, 1,3,6-Galp, t-Araf, 1,5-Araf, 1,2,5-Araf, and 1,2,4-Rhap sugar residues, suggesting that the polysaccharide samples may contain HG-type and RG-I-type pectin structural domains. The detailed connection mode was shown in Table 3.

**TABLE 3:** The sugar residue connection mode of PFPS.

Connection type	Derivative name	RT	Molar ratio
T-Ara*f*	1,4-di-O-acetyl-2,3,5-tri-O-methyl arabinitol	11.686	3.466
T-Rha*p*	1,5-di-*O*-acetyl-6-deoxy-2,3,4-tri-*O*-methyl rhamnitol	12.708	1.641
1,3-Ara*f*	1,3,4-tri-*O*-acetyl-2,5-di-*O*-methyl arabinitol	14.500	0.824
1,5-Ara*f*	1,4,5-tri-O-acetyl-2,3-di-O-methyl arabinitol	15.425	4.618
T-Gal*p*A	1,5-di-O-acetyl-2,3,4,6-tetra-O-methyl galactitol	17.449	5.763
1,3,5-Ara*f*	1,3,4,5-tetra-*O*-acetyl-2-*O*-methyl arabinitol	17.954	1.774
1,2,4-Rha*p*	1,2,4,5-tetra-*O*-acetyl-6-deoxy-3-*O*-methyl rhamnitol	18.381	1.481
1,4-Gal*p*A	1,4,5-tri-*O*-acetyl-2,3,6-tri-*O*-methyl galactitol	19.849	68.706
1,4-Gal*p*	1,4,5-tri-O-acetyl-2,3,6-tri-O-methyl galactitol	19.849	2.255
1,2,3,5-Ara*f*	1,2,3,4,5-penta-O-acetyl arabinitol	20.011	1.548
1,3-Gal*p*	1,3,5-tri-O-acetyl-2,4,6-tri-O-methyl galactitol	20.308	1.286
1,6-Gal*p*	1,5,6-tri-O-acetyl-2,3,4-tri-O-methyl galactitol	21.538	0.923
1,3,4-Gal*p*A	1,3,4,5-tetra-*O*-acetyl-2,6-di-*O*-methyl galactitol	21.952	0.844
1,2,4-Glc*p*A	1,2,4,5-tetra-*O*-acetyl-3,6-di-*O*-methyl glucitol	22.547	0.718
1,4,6-Gal*p*A	1,4,5,6-tetra-*O*-acetyl-2,3-di-*O*-methyl galactitol	23.517	0.708
1,3,6-Gal*p*	1,3,5,6-tetra-*O*-acetyl-2,4-di-*O*-methyl galactitol	24.371	3.445

**FIGURE 3:**
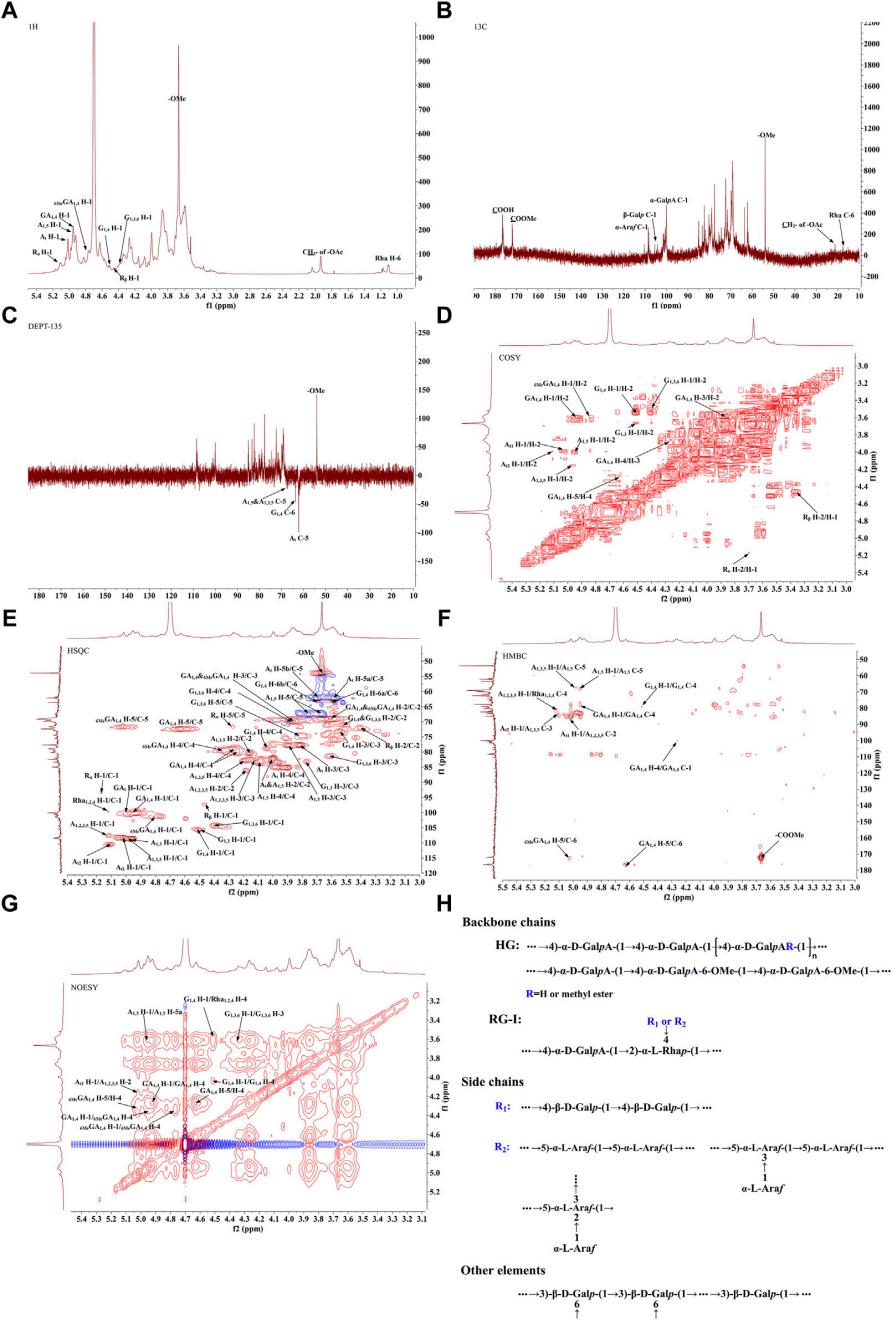
The NMR analysis of PFPS **(A)** 1H NMR spectra of PFPS. **(B)** 13C NMR spectra of PFPS. **(C)** DEPT-135 spectra of PFPS. **(D)** COSY spectra of PFPS. **(E)** HSQC spectra of PFPS. **(F)** HMBC spectra of PFPS. **(G)** NOESY spectra of PFPS. **(H)** The structure of PFPS.

The 1H-NMR spectrum (Figure 3A) showed proton resonance signals concentrated in the region of δ 3.0–5.5 ppm and δ 4.5–5.5 ppm, suggesting the presence of a variety of sugar residues. 13C-NMR spectrum (Figure 3B) showed the presence of reduced telomeric end-groups at δ 93.52 ppm and δ 97.39 ppm, which were presumed to belong to the C-1 signal peaks of the reduced telomeric end-groups α-GalpA (R_α_) and β-GalpA (R_β_), respectively. The 13C-NMR spectrum showed a significant inverted signal in the region of δ 60–70 ppm, suggesting the presence of a variety of sugar residues. The DEPT-135 spectrum (Figure 3C) revealed the presence of inverted signals in the region of δ 60–70 ppm, indicating that the sugar residues contain -CH2- groups.

The resonance signals in the region of δ 1.10–1.27 ppm revealed the presence of methyl protons H-6 of rhamnose (Rha) residues. δ 1.90–2.08 ppm showed the methyl proton signals of the O-acetyl group, and δ 21.25 ppm showed the methyl signals of the acetyl group. The signal at δ 176.44 ppm represented the characteristic absorption peak at the C-6 position in the unesterified galacturonic acid residue, and the corresponding characteristic absorption peak at δ 53.99 ppm represented the characteristic absorption peak at the C-6 position in the esterified galacturonic acid residue. The peak at δ 53.99 ppm represents the methyl carbon attached to the C-6 position in the methylated galacturonic acid residue (Figure 3D). The δ 98–102 ppm signal is presumed to be the hetero-capsule carbon of α-GalpA, and the δ 106–110 ppm is presumed to be the hetero-capsule carbon of the sugar residue α-Araf.The hetero-head signals δ 4.94 ppm (H-1) and δ 100.15 ppm (C-1) of the GA_1,4_ sugar residue were determined as α-configuration from the HSQC and 1H-1H COSY (Figure 3E). 1H-1H COSY showed the signals of H-2, H-3, and H-4, attributed to δ 3.60, δ 3.86, and δ 4.27 ppm, respectively. The chemical shifts of C-1 to C-4 on the sugar ring were determined to be δ 100.15, δ 69.23, δ 69.87, and δ 79.03 ppm, respectively. The chemical shift of C-6 was found to be δ 176.44 ppm in the HMBC spectrum (Figure 3F), which is the characteristic absorption peak in unesterified galacturonic acid residues. Combined with the results of methylation analysis and literature reports (Figures 3A–G), the GA_1,4_ sugar residue was inferred to be →4)-α-D-GalpA-(1→. The hetero-head signals δ 4.77 ppm (H-1) and δ 101.44 ppm (C-1) were found based on HSQC and 1H-1H COSY, indicating that the _6Me_GA_1,4_ is in the α-configuration. Concerning the results of the monosaccharide composition test and methylation analysis, the residue was hypothesized to be an α-GalpA sugar residue. The H-1, H-2, H-3, and H-4 signals were attributed to δ 4.77, δ 3.59, δ 3.86, and δ 4.33 ppm, respectively. The chemical shifts of C-1∼C-4 on the sugar ring as δ 101.44, δ 69.03, δ 69.87, and δ 80.02 ppm, respectively. The existence of a correlation cross-peak between C-6 and methyl proton signals of methyl ester δ 3.67 ppm δ 3.67/172.09 ppm, indicating methylation of the sugar residue. The presence of a correlation cross peak δ 3.67/172.09 ppm between C-6 and methyl proton signals of methyl ester indicated that there was methylation of the sugar residue. The chemical shifts of C-1 and C-4 were shifted towards the lower field, indicating the substitution of the residue at the C-1 and C-4 positions of the sugar ring. Combining the methylation results and literature reports, the _6Me_GA_1,4_ sugar residue was deduced to be →4)-α-GalpA-6-OMе-(1→. Following a similar approach, the major sugar residues H and C chemical shifts were summarized in Table 4.Combining the above results, the backbone chains of the PFPS were deduced as HG-type and RG-I-type pectin structural domains as well as side chains of R1 and R2 (Figure 3H).

**TABLE 4:** Chemical shift assignment of ^1^H and^13^C of each sugar residue.

Sugar residues		δ (ppm)							
			1	2	3	4	5a/5b	6a/6b	-OMe
GA_1,4_	→4)-α-D-Gal*p*A-(1→	H	4.94	3.60	3.86	4.27	4.63		
		C	100.15	69.23	69.87	79.03	72.38	176.44	
_6Me_GA_1,4_	→4)-α-D-Gal*p*A-6-OMe-(1→	H	4.77	3.59	3.86	4.33	5.03/4.96		3.67
		C	101.44	69.03	69.87	80.02	71.63	172.09	53.99
GA_t_	α-D-Gal*p*A-(1→	H	4.99	3.61	3.87	4.13	4.71		
		C	100.54	69.34	69.66	71.85	72.42	176.32	
G_1,4_	→4)-β-D-Gal*p*-(1→	H	4.51	3.52	3.54	4.04	3.64	3.59/3.69	
		C	105.59	71.08	73.05	78.74	74.41	63.57	
G_1,3,6_	→3,6)-β-D-Gal*p*-(1→	H	4.39	3.51	3.61	3.85	3.81	3.79/3.93	
		C	104.27	70.84	81.28	70.14	74.56	69.57	
G_1,3_	→3)-β-D-Gal*p*-(1→	H	4.50	3.65	3.77	—	—	—	
		C	105.44	71.19	83.06	—	—	—	
A_1,5_	→5)-α-L-Ara*f*-(1→	H	4.95	3.99	3.89	4.08	3.67/3.75		
		C	108.51	82.32	77.81	83.34	67.60		
A_1,3,5_	→3,5)-α-L-Ara*f*-(1→	H	4.97	4.16	3.96	4.18	3.70/3.82		
		C	108.56	80.35	83.49	82.71	67.36		
A_t1_	α-L-Ara*f*-(1→	H	5.02	3.99	3.82	3.90	3.58/3.69		
		C	108.21	82.32	77.71	85.07	62.15		
A_t2_	α-L-Ara*f*-(1→	H	5.11	3.99	3.82	3.90	3.58/3.69		
		C	110.47	82.32	77.71	85.07	62.15		
A_1,2,3,5_	→2,3,5)-α-L-Ara*f*-(1→	H	5.12	4.18	4.12	4.18	3.70/3.82		
		C	107.57	86.10	82.38	82.46	67.36		
Rha_1,2,4_	→2,4)-α-L-Rha*p*-(1→	H	5.12	4.00	3.92	3.57	3.81	1.12	
		C	99.34	77.41	70.84	81.59	71.63	17.65	
R_α_	→4)-α-D-Gal*p*A	H	5.16	3.69	3.83	4.43	4.27	--	
		C	93.52	69.26	69.87	79.94	71.48	--	
R_β_	→4)-β-D-Gal*p*A	H	4.47	3.37	3.63	4.24	--	--	
		C	97.39	72.66	73.86	80.09	--	--	

### 3.2 PFPS inhibited LPS-induced activation of RAW264.7 cells

**FIGURE 4:**
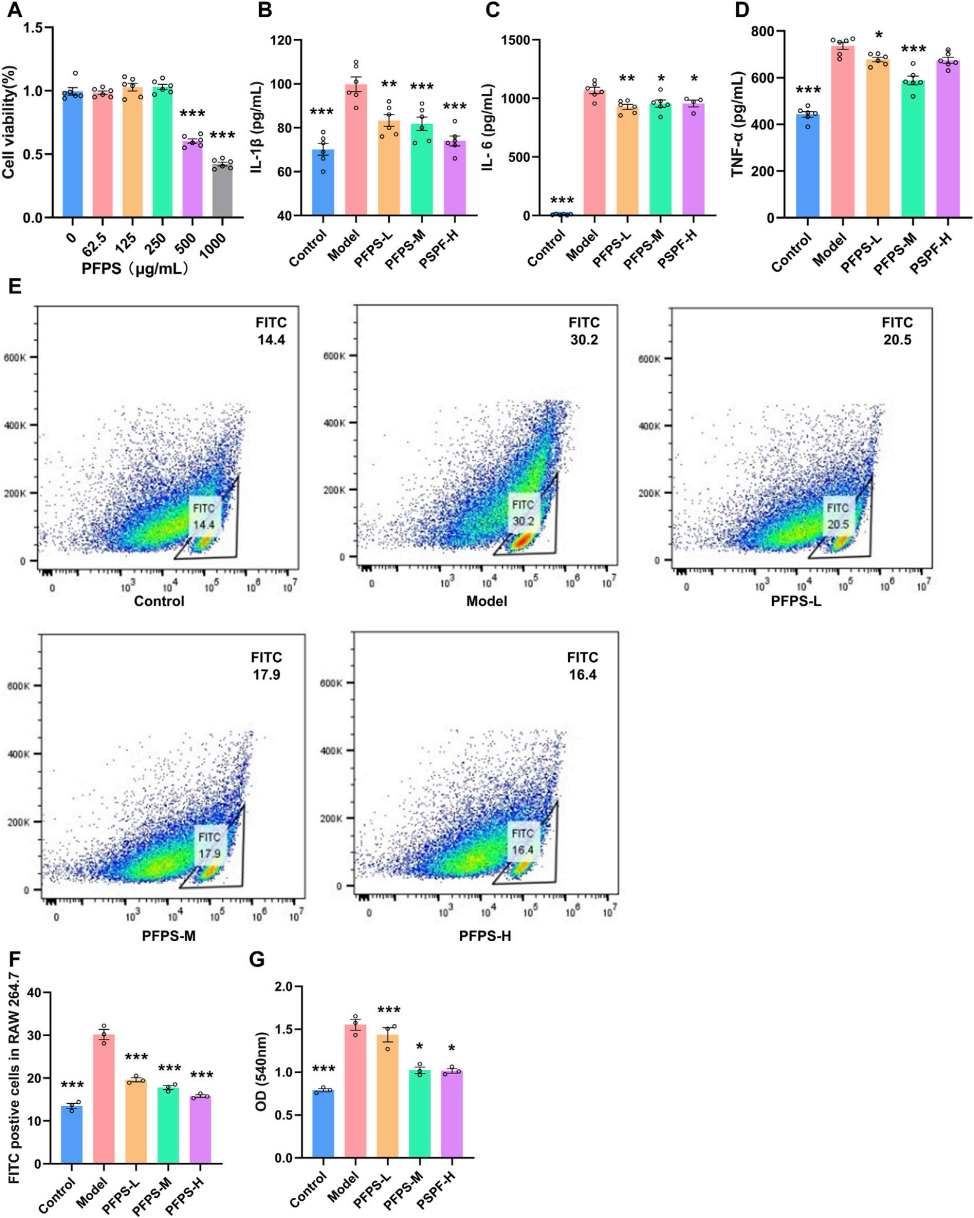
PFPS improved inflammation in LPS-induced RAW264.7 cells. **(A)** Cell activity detecting results of RAW264.7 cells treated with PFPS, n = 6. **(B–D)** The content of IL-1β **(B)**, IL-6 **(C)**, and TNF-α **(D)** in all groups, n = 6. **(E, F)** Phagocytosis of *Escherichia coli* by RAW 264.7 cells, *Escherichia coli* was marked by FITC, n = 3. **(G)** Phagocytosis of neutral red by RAW264.7 cells, n = 3. The data represent the means ± SEM. *: *p* < 0.05, **: *p* < 0.01, ***: *p* < 0.001, vs. Model group.

Our study preliminarily evaluated the anti-inflammatory efficacy of PFPS on LPS-induced RAW264.7 cells. As shown in Figure 4A, RAW264.7 cell viability decreased under PFPS treatment at doses higher than 250 μg/mL. Therefore, 62.5, 125, and 250 μg/mL PFPS were selected for following experiments, and the corresponding treatment groups were named PSPF-L, PFPS-M, and PFPS-H respectively. Compared with the Model group, PFPS inhibited the release of IL-1β, IL-6, and TNF-α from LPS-induced RAW264.7 cells (Figures 4B–D). As shown in Figures 4E, F, the phagocytosis of *E. coli* by cells in the Model group was Significantly increased, while PFPS intervention significantly reduced phagocytosis of RAW246.7 cells. And PFPS reduced LPS-induced neutral red uptake (Figure 4G). Therefore, PFPS significantly inhibited the phagocytosis activity in LPS-induced RAW264.7 cells. In summary, our results show that PFPS can significantly improve LPS-induced inflammation in RAW264.7 cells.

### 3.3 PFPS suppressed inflammation in mouse ear edema and mouse paw edema models

**FIGURE 5:**
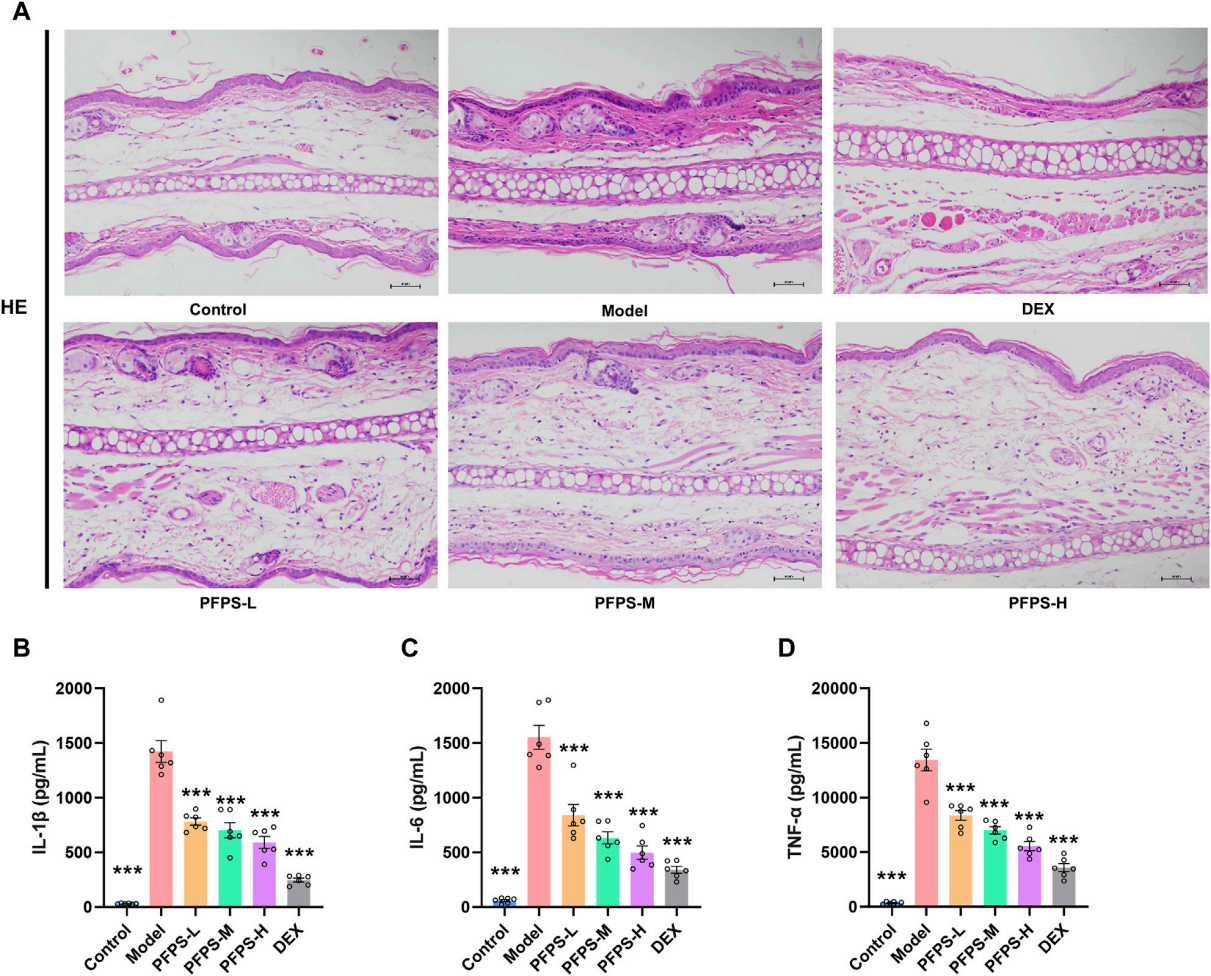
PFPS impoved ear edema inflammatory model. **(A)** The pathological observatiom of ear tissue of mice in each group (HE, x200). **(B–D)** The content of IL-1β **(B)**, IL-6 **(C)**, TNF-α **(D)** in ear tissue, n = 6. The data represent the means ± SEM. *: *p* < 0.05, **: *p* < 0.01, ***: *p* < 0.001, vs. Model group.

This study used a xylene-induced mouse ear swelling model to evaluate the *in vivo* anti-inflammatory efficacy of PFPS. The results of pomegranate flower polysaccharide on xylene-induced auricle swelling in mice are shown in Table 5. Compared with the Control group, the swelling degree of the Model group increased significantly, indicating that the modeling was successful. Compared with the model group, both the PFPS-treated groups and the DEX group significantly reduced the swelling of the auricles induced by xylene inflammation. The PFPS-H group had the best anti-inflammatory effect, which was similar to that of the DEX group. The results of the ear tissue pathological sections are shown in Figure 5A. The Model mouse ears exhibited edema, the gaps between collagen fibers increased, and inflammatory cells infiltrated, accompanied by congestion, hemorrhage, and necrosis of tissue cells. Compared with the Model group, the inflammatory cells in the DEX group were significantly reduced but accompanied by edema, congestion, and hemorrhage. In the PFPS supplement group, as the dose increased, related symptoms continued to decrease. In all groups, the PFPS-H group had the best effect, with the fewest inflammatory cells and the least congestion. Consistent with *in vitro* experiments, the level of IL-1β, IL-6, and TNF-α in ear tissue were also significantly downregulated affected by PFPS (Figures 5B–D). Therefore, PFPS reduced xylene-induced ear swelling in mice by improving cytokine secretion. Further, our experiments tested the inhibitory effect of PFPS on mouse foot swelling. As the Table 6 showed that different doses of PFPS had a certain inhibitory effect on foot swelling, and the PFPS-H group had the best anti-inflammatory effect. The results of *in vivo* and *in vitro* experiments show that PFPS has significant anti-inflammatory effects.

**TABLE 5:** Determination of ear edema degree of mice in each group.

Group	Dosage	Inhibition rate (%)	Swelling rate (%)
Control	saline	—	0.001433 ± 0.00132***
Model	saline	—	0.012816 ± 0.00216
PFPS-L	400 mg/kg	15.0033	0.010894 ± 0.00193*
PFPS-M	800 mg/kg	26.5930	0.009408 ± 0.00143**
PFPS-H	1,600 mg/kg	41.8076	0.007458 ± 0.00184**
DEX	10 mg/kg	43.9532	0.007183 ± 0.00117**

Note: *: *p* < 0.05, **: *p* < 0.01, ***: *p* < 0.001, vs. Model group.

**TABLE 6:** Determination of mouse Paw Edema in each group.

Group	0 h	1 h	2 h	3 h	4 h	5 h	6 h
Model	1.05 ± 0.11	1.00 ± 0.31	1.36 ± 0.15	1.75 ± 0.22	1.37 ± 0.16	1.23 ± 0.13	1.67 ± 0.13
PFPS-L (400 mg/kg)	0.99 ± 0.21	0.74 ± 0.32	1.20 ± 0.17	1.32 ± 0.18	1.36 ± 0.15	1.16 ± 0.07	0.97 ± 0.18^*^
PFPS-M (800 mg/kg)	1.03 ± 0.27	0.96 ± 0.39	0.86 ± 0.12^**^	1.09 ± 0.19^**^	1.15 ± 0.26	0.96 ± 0.07^*^	0.84 ± 0.07^*^
PFSP-H (1600 mg/kg)	1.02 ± 0.12	1.09 ± 0.27	0.86 ± 0.22^**^	1.01 ± 0.13^**^	0.90 ± 0.20^**^	0.84 ± 0.12^**^	0.80 ± 0.21^**^
DEX (5 mg/kg)	0.98 ± 0.17	0.73 ± 0.26	0.77 ± 0.14^**^	0.90 ± 0.21^**^	0.67 ± 0.19^**^	0.64 ± 0.17^**^	0.59 ± 0.08^***^

Note: The data represent the means ± SEM. *: *p* < 0.05, **: *p* < 0.01, ***: *p* < 0.001, vs. Model group.

### 3.4 PFPS improved LPS-induced mastitis in mice

**FIGURE 6:**
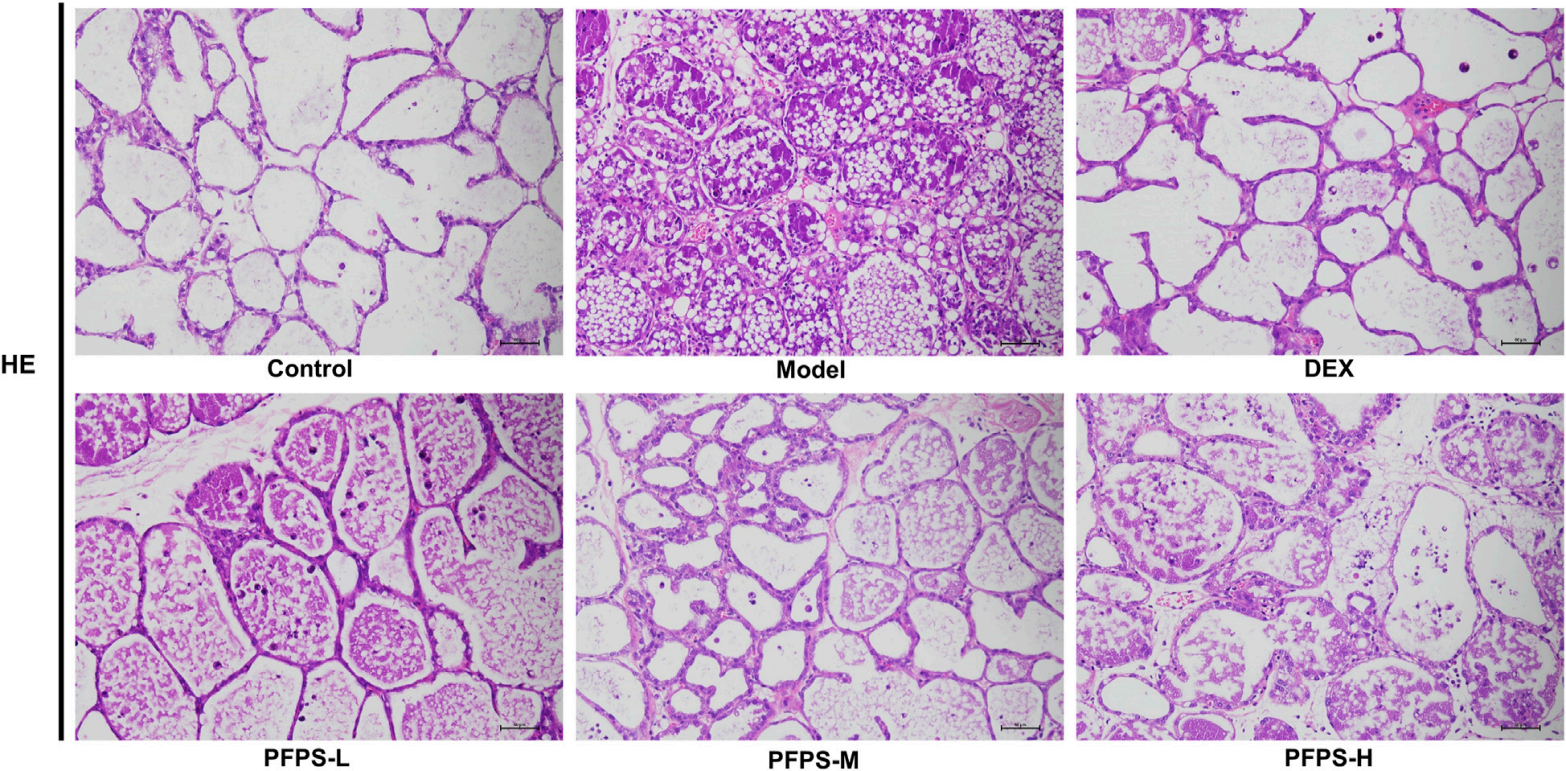
HE staining results of mouse breast tissue in all groups (HE, x200).

**FIGURE 7:**
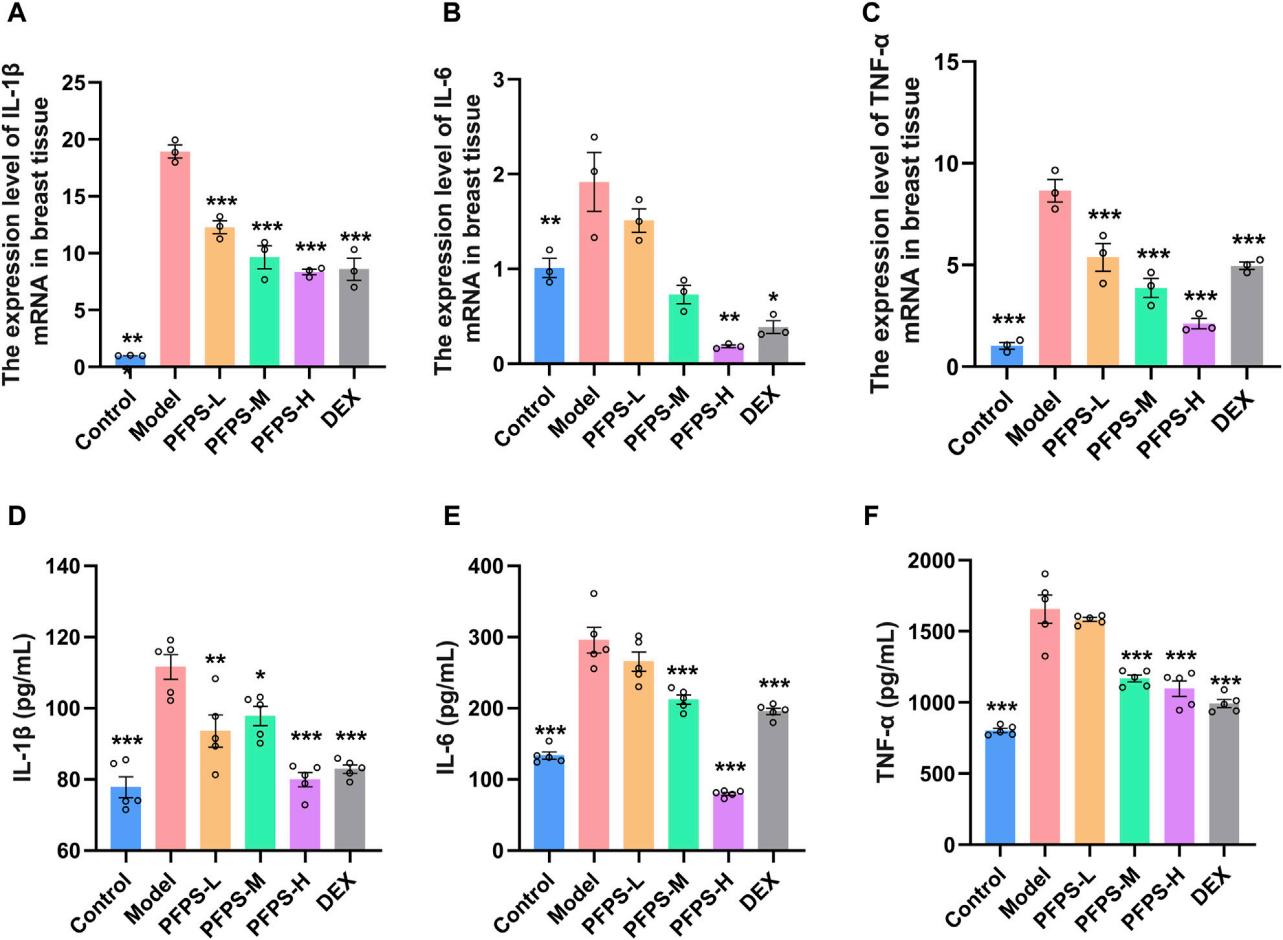
**(A–C)** The expression level of IL-1β **(A)**, IL-6 **(B)**, and TNF-α **(C)** mRNA in breast tissue, n = 3. **(D–E)** The content of IL-1β **(D)**, IL-6 **(E)**, and TNF-α **(F)** in breast tissue, n = 5. The data represent the means ± SEM. *: *p* < 0.05, **: *p* < 0.01, ***: *p* < 0.001, vs. Model group.

Our study evaluated the *in vivo* anti-inflammatory effects of PFPS using an LPS-induced mouse mastitis model. As shown in Figure 6, PFPS dose-dependently improved mastitis in mice. Compared with the Control group, the Model group had disordered alveolar structure, widened breast lobule interstitium, increased inflammatory cells in the alveoli, and vascular congestion, which indicated that LPS modeling was successful. Compared with the Model group, the number of inflammatory cells in the DEX group was significantly reduced, with less inflammatory cell infiltration and mild congestion. There was inflammatory cell infiltration in all PFPS medication groups, among which PFPS-H had the best effect. To clarify the anti-inflammatory effect of PFPS, our experiment further detected the content of pro-inflammatory cytokines and the corresponding mRNA expression levels in mouse breast tissue. As shown in Figures 7A–F, PFPS significantly inhibited the mRNA expression levels of IL-1β, IL-6, and TNF-α in the mammary tissue of model mice, and PFPS significantly reduced the contents of IL-1β, IL-6, and TNF-α in breast tissue. Therefore, PFPS can significantly improve LPS-induced mammary gland inflammation in mice.

### 3.5 PFPS improved LPS-induced oxidative stress in mouse mammary gland tissue

**FIGURE 8:**
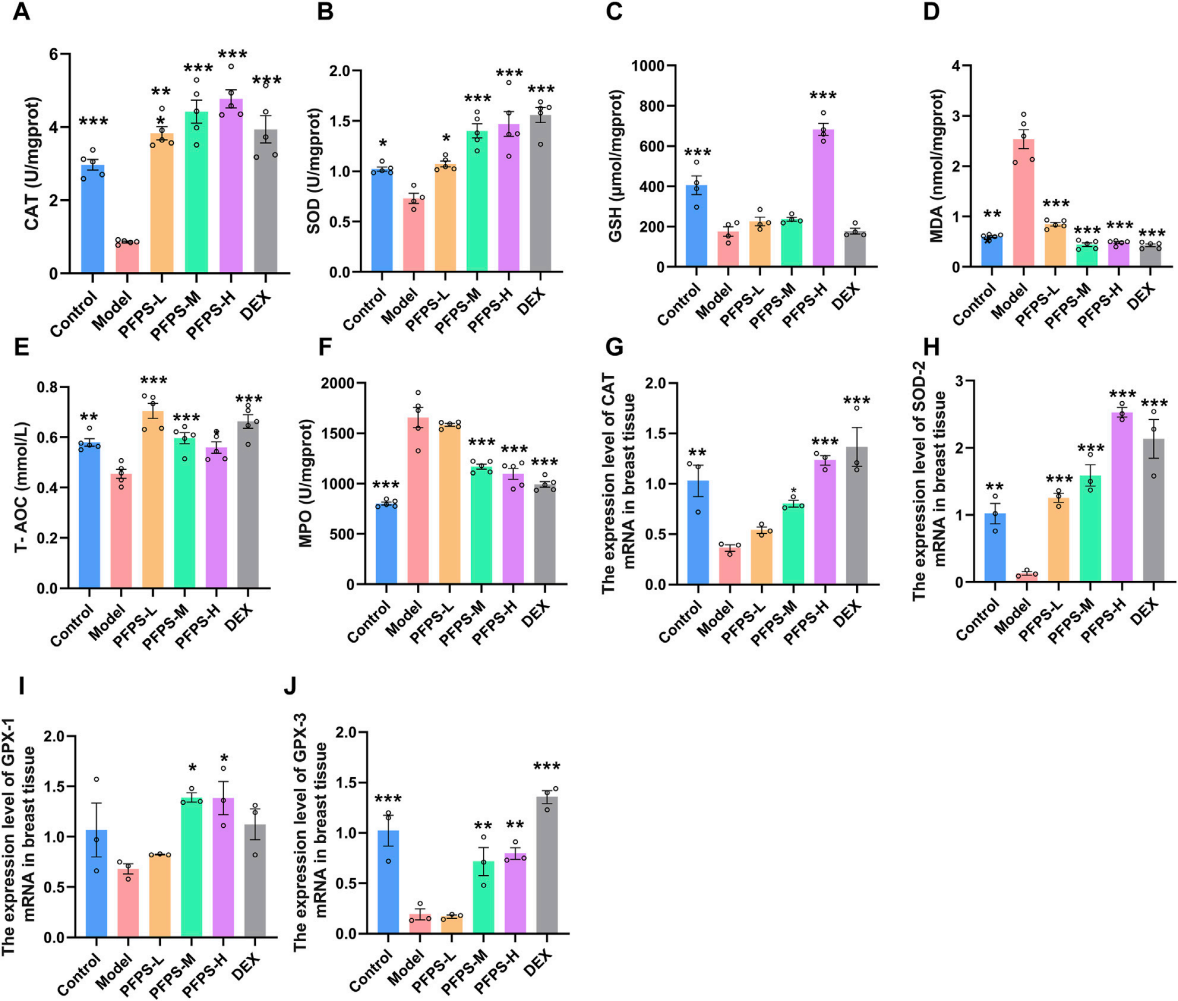
PFPS improves LPS-induced oxidative stress in mouse mammary gland tissue. **(A–F)** The content of CAT **(A)**, SOD **(B)**, GSH **(C)**, MDA **(D)**, MPO **(E)**, T-AOC **(F)** in mouse mammary gland tissue, n = 5. **(G–H)** the expression level of CAT **(G)**, SOD-2 **(H)**, GPX-1 **(I)**, GPX-3 **(J)** mRNA in breast tissue, n = 3. The data represent the means ± SEM. *: *p* < 0.05, **: *p* < 0.01, ***: *p* < 0.001, vs. Model group.

LPS causes oxidative stress in inflammatory tissues, so oxidative stress-related indicators in mice breast tissue were detected. PFPS supplementation significantly improved the LPS-induced decrease in the levels of CAT, SOD, GSH, and T-AOC and the increase in MDA levels in breast tissue, and the PFPS high-dose group had the best effect (Figures 8A–E). PFPS also reduced MPO content in breast tissue (Figure 8F). PCR results showed that PFPS could significantly increase the mRNA expression of antioxidant-related enzymes in breast tissue (Figures 8G–J). Therefore, PFPS can significantly improve the oxidative stress in LPS-induced breast tissue in a dose-dependent manner.

### 3.6 PFPS effectively modulated intestinal flora in LPS-modeling mice

**FIGURE 9:**
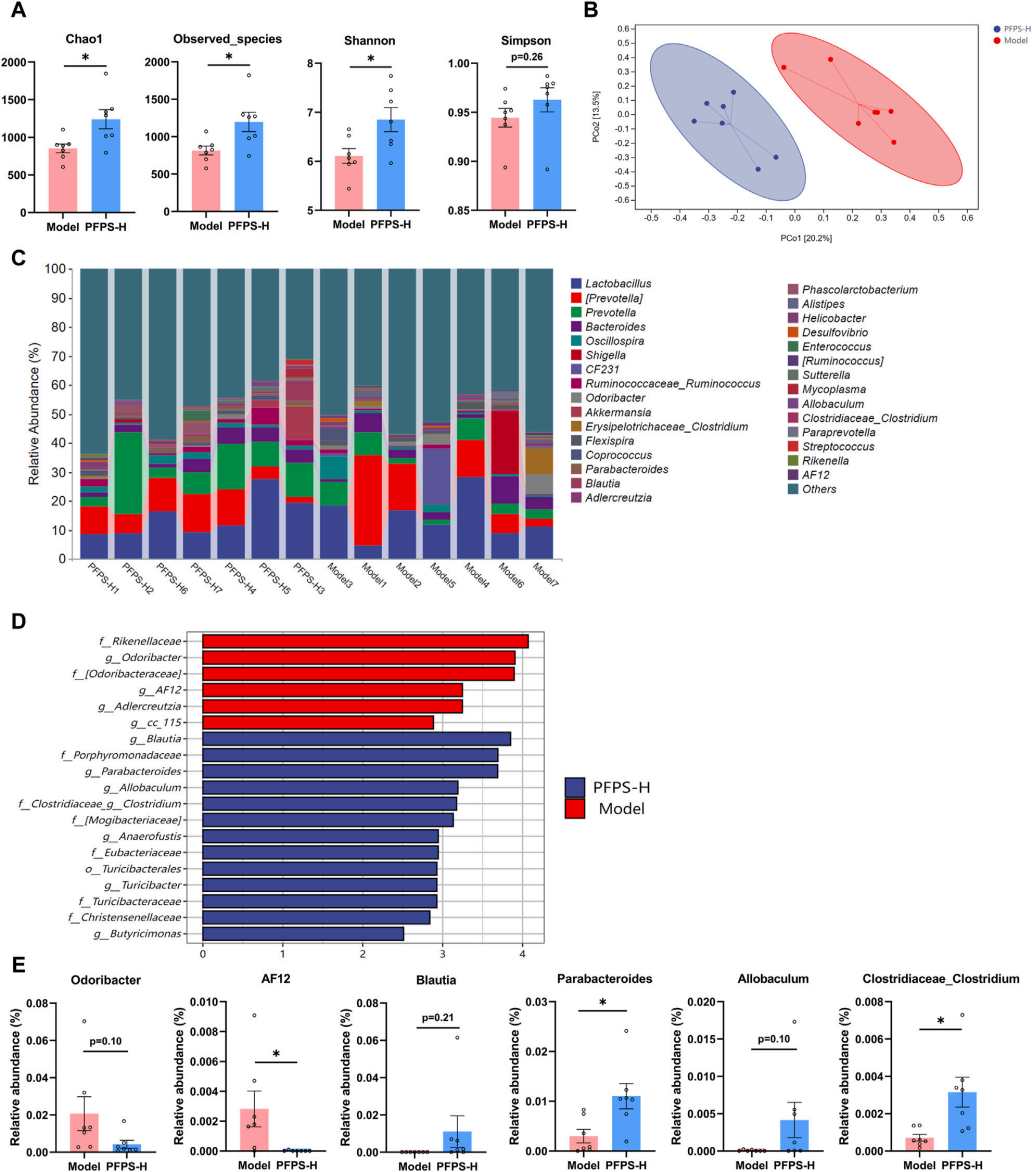
PFPS changed the composition of gut flora; n = 7. **(A)** α-analyses indices (Chao1, Observed_species, Shannon, and Simpson). **(B)** PCoA plot of the gut microbiota based on bray_curtis distance. **(C)** Top 30 microbiota composition at levels of genus. **(D)** Diagram of LEfSe (LDA > 4.0). **(E)** The relative abundance of key genera: *Odoribacter*, *AF12*, *Blautia*, *Parabacteroides*, *Allobaculum*, and *Clostridiaceae_Clostridum*. The data represent the means ± SEM. *: *p* < 0.05, **: *p* < 0.01, ***: *p* < 0.001, vs. Model group.

As shown in Figure 9A, supplementation with PFPS significantly increased the richness and diversity of the intestinal microbiota in mice. The PCOA plot based on the bray_curtis distance algorithm showed that the PFPS and Model groups were significantly separated (Figure 9B). Figure 9C shows the composition of the top 30 bacterial groups with relative abundance in each sample. Combined with the LDA discrimination chart shown in Figure 9D, the biomarkers under PFPS intervention were screened out, and their specific relative abundances are shown in Figure 9E. PFPS can suppress the relative abundance of *Odoribacter* and *AF12* and increase *Blautia*, *Parabacteroides*, *Allobaculum*, and *Clostridiaceae_Clostridium*. Therefore, PFPS can significantly modulate the composition of intestinal flora in mice.

### 3.7 PFPS improves mastitis in mice by enhancing the blood-milk barrier

**FIGURE 10:**
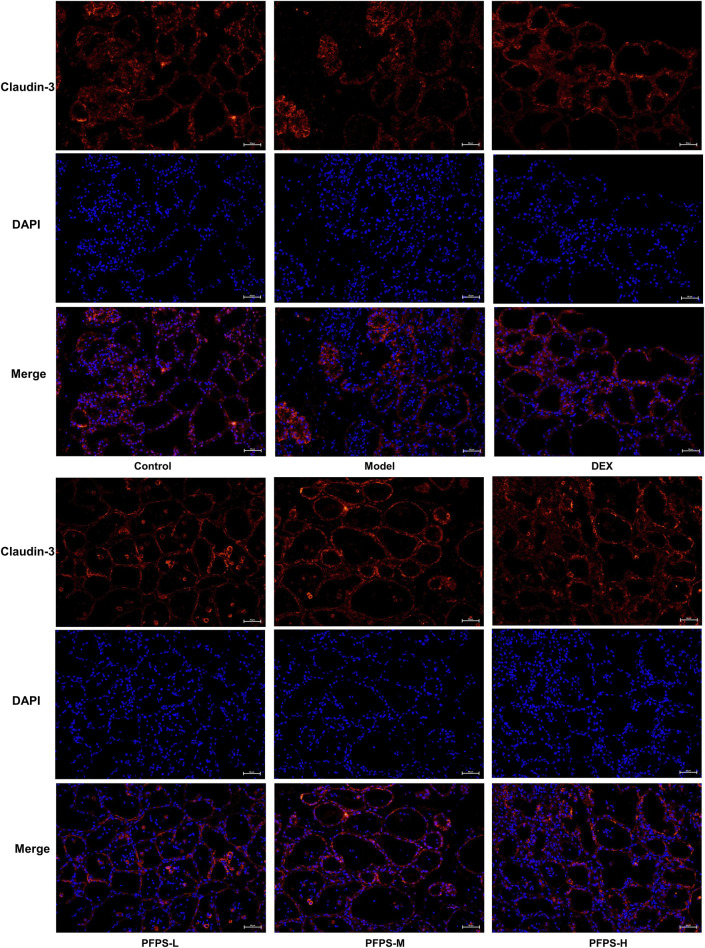
Immunofluorescence staining of Claudin-3 in breast tissue (x200).

**FIGURE 11:**
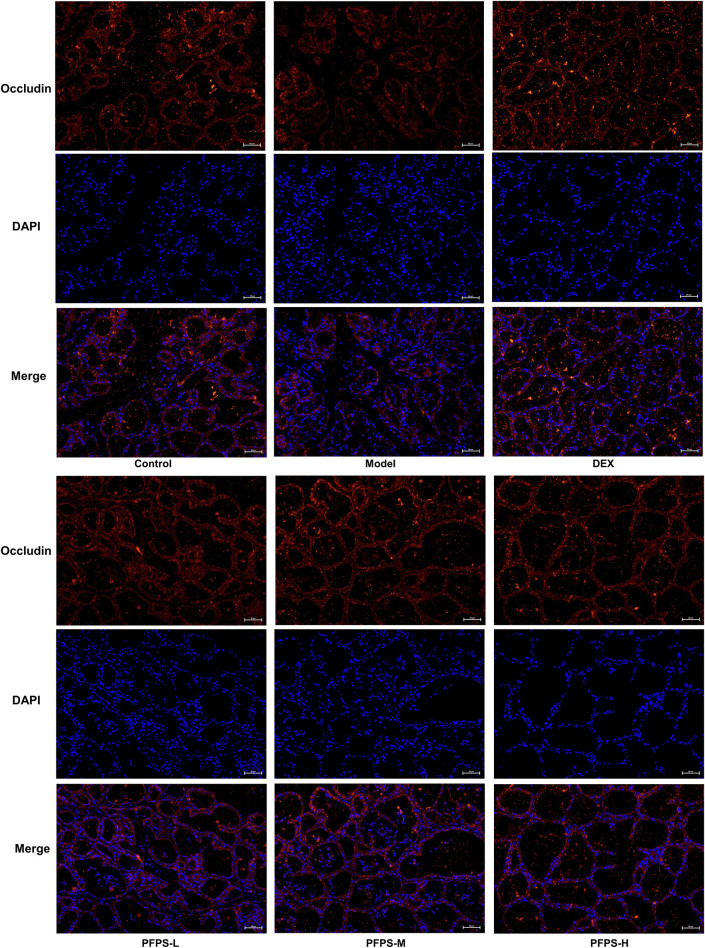
Immunofluorescence staining of Occludin in breast tissue (x200).

**FIGURE 12:**
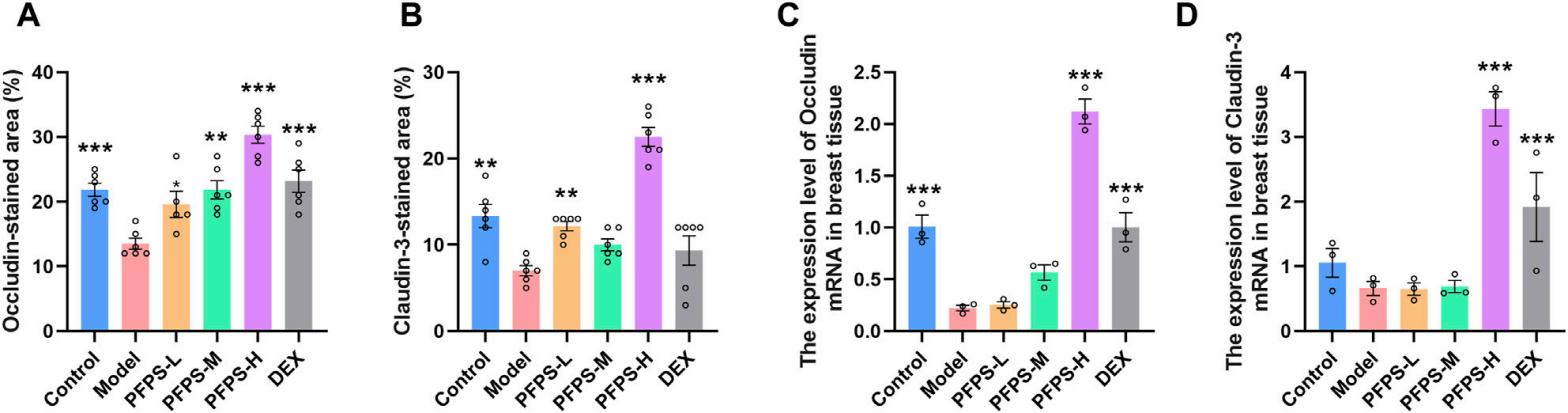
PFPS restored tight junctions in the breast tissue. **(A, B)** Quantification of relative protein expressions, n = 6. **(C, D)** Quantification of mRNA expressions, n = 3. The data represent the means ± SEM. *: *p* < 0.05, **: *p* < 0.01, ***: *p* < 0.001, vs. Model group.

This experiment used immunofluorescence to measure the integrity of the blood-milk barrier. As shown in Figures 10–12, LPS reduced the expression of barrier proteins Claudin-3 and Occludin, while the PFPS supplementation group and the DEX group antagonized the effects of LPS. Quantitative staining area results showed that PFPS could significantly increase the content of barrier proteins in LPS model mice, and the PFPS-H group had the best effect. PFPS-H caused high expression of Claudin-3 and Occludin, and their values were significantly higher than those in the blank and DEX groups. The expression levels of related genes were further tested and a consistent change trend was obtained. Therefore, PFPS can improve mouse mastitis by enhancing the blood-milk barrier.

## 4 Discussion

The PFPS was isolated and purified by ion-exchange column chromatography and gel column chromatography, and its chemical structure was characterized by using a combination of technique including infrared spectroscopy, liquid chromatography, gas chromatography, and NMR. The result revealed that the monosaccharides consist of GalA, Ara, and Gal, and the residues consist of 1,4-GalpA, 1,4-Galp, and 1,3,6-Galp, which may contain HG-type and RG-I-type pectin structural domains. Similar methods have been validated for the identification of plant polysaccharides. For example, the chemical structure of licorice polysaccharide was identified and characterized by NMR and methylation analysis, and the result revealed that GPS-1 is composed of homogalacturonan (HG)-type pectin with 4)-D-GalpA-(1 as the backbone (Wu et al., 2022). Polysaccharide BAP1 of Bifidobacterium adolescentis CCDM 368 consists of glucose, galactose, and rhamnose residues and was deduced by methylation analysis and NMR spectrum (Pacyga-Prus et al., 2023). The above results demonstrate the scientific validity of methylation and spectroscopy in elucidating the structure and composition of polysaccharides.

This experiment established an *in vitro* cell inflammation model by stimulating RAW264.7 cells with LPS. Macrophages are important immune cells involved in the initiation of inflammatory responses and play a key role in the pathogenesis of numerous inflammatory diseases by secreting large amounts of pro-inflammatory mediators and pro-inflammatory cytokines (Wynn and Vannella, 2016; Oishi and Manabe, 2018). We found that PFPS can significantly inhibit the release of pro-inflammatory factors of RAW264.7 cells induced by LPS. Past research shows the release of pro-inflammatory factors increases significantly when inflammation occurs (Huang et al., 2023). LPS can promote the secretion of cytokines TNF-α, IL-6, and IL-1β (Wang et al., 2020). As intercellular signaling molecules, these factors can attract neutrophils in the blood into the infected area to eliminate pathogens, but they can also cause damage to breast tissue, resulting in cell necrosis and suppuration (O'Neill et al., 2016). Experiment results showed that PFPS also significantly inhibited the phagocytosis of neutral red dye and *E. coli* by RAW264.7 cells. Macrophages present antigens through phagocytosis, and LPS can induce the enhancement of phagocytosis of RAW264.7 cells by regulating the JNK signaling pathway and promoting the occurrence of inflammation (Yuan et al., 2019; Chen et al., 2021). In summary, PFPS exhibits significant inflammation-inhibitory effects in vitro models.

This experiment preliminarily evaluated the *in vitro* inflammation inhibitory effect of PFPS through mouse ear swelling and foot swelling models. The ear swelling model is widely used in evaluation experiments of anti-inflammatory drugs, in which xylene induces the ear swelling model by activating phospholipase A2 (Santos et al., 2012). The pathological process of ear swelling is closely related to the increase in pro-inflammatory factors, such as IL-1β is considered to be the main cytokine in the initiation of acute inflammation mediated by keratinocytes (Soromou et al., 2012). The paw swelling model in mice is also a widely recognized model of inflammation *in vivo* (Zhang et al., 2022; Hijazy et al., 2023). The results showed that PFPS significantly inhibited ear swelling and foot swelling, showing a significant inhibitory effect on inflammation *in vivo*.

PFPS had shown anti-inflammatory effects in both *in vivo* and *in vitro* models, so we further evaluated the anti-inflammatory effects and potential intervention pathways of PFPS in a mouse mastitis model. LPS can induce oxidative stress in breast tissue (increased intracellular ROS, MDA, and decreased SOD, GSH-Px and CAT activity), and oxidative stress aggravates tissue inflammation by increasing the release of inflammatory factors (increased expression of IL-6, IL-Iβ, and TNF-α) (Ali et al., 2022). The results showed that high-dose PFPS significantly improved the oxidative stress in the mammary gland of model mice and antagonized the increase in the contents and mRNA expression levels of IL-1β, IL-6, and TNF-α. Research on polysaccharides shows that most polysaccharides exert systemic effects by regulating intestinal flora (Tang et al., 2019; Liu et al., 2023). At the same time, considering that PFPS has a significant inhibitory effect on inflammation in models of mastitis, ear swelling, and paw swelling, we believe that PFPS may regulate systemic inflammation by regulating intestinal flora. Sequencing results showed that PFPS significantly changed the composition of intestinal flora in model mice. PFPS decreased the relative abundance of *Oscillospira* and *AF12*. *Oscillospira* is a common opportunistic pathogen closely associated with inflammation (Xu R. et al., 2022). Zhu et al., (2020) found that Oscillospira positively correlates with diabetes and inflammation while feeding mice oat-resistant-starch can significantly inhibit Oscillospira and improve inflammation. Wei et al., (2023) found that Glycyrrhiza polysaccharide can inhibit Oscillospira and improve DSS-induced intestinal inflammation. PFPS also reduced the relative abundance of Odoribacter. Andrea et al. found that Odoribacter was positively correlated with the degree of inflammation in the body and that *Odoribacter* could cause systemic inflammation when studying neuroinflammation and intestinal flora (Quagliariello et al., 2018). PFPS increased the relative abundance of *Blautia*, *Parabacteroides*, *Allobaculum*, and *Clostridium. Blautia* is a butyrate producer and regulates the metabolic processes of animals through exogenous butyrate supplementation (Zhou et al., 2017). Related studies have shown that butyrate supplementation can alleviate LPS damage to the blood-milk barrier and reduce inflammatory responses (Wang et al., 2017a). *Parabacteroides* and *Allobaculum* are the common probiotic that has anti-inflammatory effects and produces SCFA (Cui et al., 2022; Zhang et al., 2023). Wu et al. (2019) found that supplied *Hirsutella sinensis* polysaccharide can increase the relative abundance of *Parabacteroides goldsteinii*. Supplementation of *P. goldsteinii* alone can significantly reduce the content of inflammatory factors in serum, and improve inflammation and intestinal barrier. *Allobaculum* can increase the levels of intestinal and blood-brain barrier proteins, attenuate the effects of LPS and alleviate neuroinflammation (Yuan et al., 2022). *Clostridium* also has anti-inflammatory effects. Related studies have shown that *Clostridium leptum* can induce the production of IL-10^+^ regulatory B cells to treat airway inflammation (Huang et al., 2022). Studies have shown that intestinal SCFA-producing bacteria can protect the blood-milk barrier and improve mastitis through SCFA (Hu et al., 2020). Ran et al. demonstrated that hesperidin significantly improved the blood-milk barrier by increasing acetic acid and propionic acid producing bacteria (Ran et al., 2024). Hu et al. found that supplementation with butyrate-producing bacteria significantly reduced the permeability of the blood-milk barrier and inhibited the occurrence of mastitis (Hu et al., 2020). Our results showed that PFPS significantly restored LPS-induced BMB disruption by promoting the content and mRNA expression of tight junction proteins Claudin-3 and Occludin. Therefore, PFPS exerts anti-mastitis effects by increasing the relative abundance of SCFA-producing bacteria and improving the blood-milk barrier.

## 5 Conclusion

PFPS reduced the production of proinflammatory factors and inhibited the phagocytic ability of RAW264.7 cells *in vitro*. PFPS showed significant anti-inflammatory effects *in vivo* model of mouse ear swelling, foot swelling, and mastitis. 16S RNA sequencing results showed that PFPS significantly regulated the composition of mouse intestinal flora, decreased the relative abundance of Oscillospira and AF12, and increased Blautia, Parabacteroides, Allobaculum, and *Clostridium*. Therefore, PFPS reduced LPS-induced mastitis by regulating intestinal flora and improving the blood-milk barrier. There are still many deficiencies in this article, for example: the effect of PFPS on regulating intestinal flora requires FMT experiments for inflammation; at the same time, due to the limitations of sequencing technology, the specific flora regulated by PFPS cannot be accurately determined; whether the regulatory effect of PFPS in vitro experiments can occur *in vivo* independently of regulating flora still needs to be verified by germ-free mice.

## Data Availability

The datasets presented in this study can be found in online repositories. The names of the repository/repositories and accession number(s) can be found in the article/supplementary material.
